# Relationship between Surface Properties and In Vitro Drug Release from Compressed Matrix Containing Polymeric Materials with Different Hydrophobicity Degrees

**DOI:** 10.3390/ph10010015

**Published:** 2017-01-24

**Authors:** Cristhian J. Yarce, Juan D. Echeverri, Mario A. Palacio, Carlos A. Rivera, Constain H. Salamanca

**Affiliations:** Pharmaceutical Physical Chemistry Laboratory, Natura Research Group, Pharmaceutical Chemistry Program, Faculty of Natural Sciences, ICESI University, Cali 760031, Colombia; cjyarce@icesi.edu.co (C.J.Y.); juandiegoqf@hotmail.com (J.D.E.); marioandres_8@hotmail.com (M.A.P.); carlosrive114@gmail.com (C.A.R.)

**Keywords:** contact angle, surface free energy, drug release, polymeric materials, ampicillin trihydrate

## Abstract

This work is the continuation of a study focused on establishing relations between surface thermodynamic properties and in vitro release mechanisms using a model drug (ampicillin trihydrate), besides analyzing the granulometric properties of new polymeric materials and thus establishing the potential to be used in the pharmaceutical field as modified delivery excipients. To do this, we used copolymeric materials derived from maleic anhydride with decreasing polarity corresponding to poly(isobutylene-*alt*-maleic acid) (hydrophilic), sodium salt of poly(maleic acid-*alt*-octadecene) (amphiphilic), poly(maleic anhydride-*alt*-octadecene) (hydrophobic) and the reference polymer hydroxyl-propyl-methyl-cellulose (HPMC). Each material alone and in blends underwent spectroscopic characterization by FTIR, thermal characterization by DSC and granulometric characterization using flow and compaction tests. Each tablet was prepared at different polymer ratios of 0%, 10%, 20%, 30% and 40%, and the surface properties were determined, including the roughness by micro-visualization, contact angle and water absorption rate by the sessile drop method and obtaining W_adh_ and surface free energy (SFE) using the semi-empirical models of Young–Dupré and  Owens-Wendt-Rabel-Käelbe (OWRK), respectively. Dissolution profiles were determined simulating physiological conditions in vitro, where the kinetic models of order-zero, order-one, Higuchi and Korsmeyer–Peppas were evaluated. The results showed a strong relationship between the proportion and nature of the polymer to the surface thermodynamic properties and kinetic release mechanism.

## 1. Introduction

**Figure 1:**
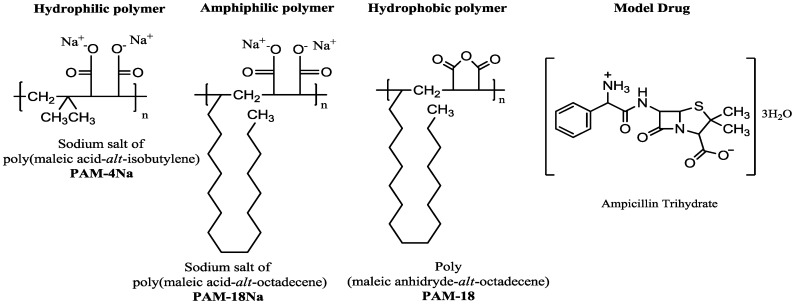
Chemical structure of the monomer units of polymers derived from maleic anhydride and ampicillin trihydrate, corresponding to the materials used in the study.

The development of new devices for controlled release of drugs corresponds to a large area of interest in the fields of chemistry and pharmacy [1,2]. In this sense, different matrix systems of a polymeric character are used as a viable alternative with great potential for application in the design and formulation of pharmaceutical dosage forms [3,4,5]. Currently, many polymeric materials have been used for this purpose, where the polymers derived from maleic anhydride are a worthwhile alternative, since they have shown great potential as controlled drug delivery systems [6,7,8,9,10,11,12]. This class of polymers has attracted much attention due to the characteristics of biocompatibility, clearly defined structure and versatility to combine with other precursors to obtain materials with multiple properties and applications [13,14]. Another feature presented by those copolymers is that the maleic anhydride ring may break, leading to the formation of two carboxylic acids that enhance the solubility in aqueous media [15,16], while the other co-monomeric unit may confer different properties to the polymer. Therefore, those materials project a great potential to become excipients for the pharmaceutical sector. Some of these polymeric materials derived from maleic anhydride are poly(maleic anhydride-*alt*-octadecene), referred to as PAM-18, poly(isobutylene-*alt*-maleic acid) and the sodium salt of poly(maleic acid-*alt*-octadecene), known as PAM-4Na and PAM-18Na, respectively, which are shown in Figure 1. The PAM-18Na polymer has several interesting properties, such as the ability to abruptly lower the surface tension of water [17] and form hydrophobic pseudo-phases of the intra- and inter-molecular type depending on its concentration, establishing reservoir systems for drug vehiculization. Additionally, there are other studies that indicate the potential usage for PAM polymers in the preparation of compressed matrix systems containing drugs, because of the physicochemical interactions between polymeric materials and drugs that could be able to modify several characteristics from hardness, water absorption rates, surface properties, to drug release profiles.

It is worthy to note that although many studies have described drug delivery using polymeric materials, there are very few studies relating the release mechanisms from compressed tablets with the hydrophobicity degree of the polymeric materials and the surface properties of the tablets [18,19,20]. For this reason, this work is the continuation of a study focused on establishing relations between the surface thermodynamic properties and in vitro release mechanisms using a model drug (ampicillin trihydrate) as the model and similar polymeric materials with different degrees of hydrophobicity, such as PAM-4Na, PAM-18Na and PAM-18. In addition, we used hydroxyl-propyl-methyl-cellulose (HPMC), a polymeric material of reference and widely used in studies of modified release [21,22,23]. Finally, it should be explained that we used ampicillin trihydrate as a model drug (Figure 1), because the drug has a rapid rate of dissolution in the physiological media, thus allowing us to relate changes in the dissolution profiles associated with a modified release given by an effect of the polymeric matrix and not a low intrinsic dissolution effect [15,24].

## 2. Results and Discussion

### 2.1. Preparation and Characterization of Copolymers Materials

**Figure 2:**
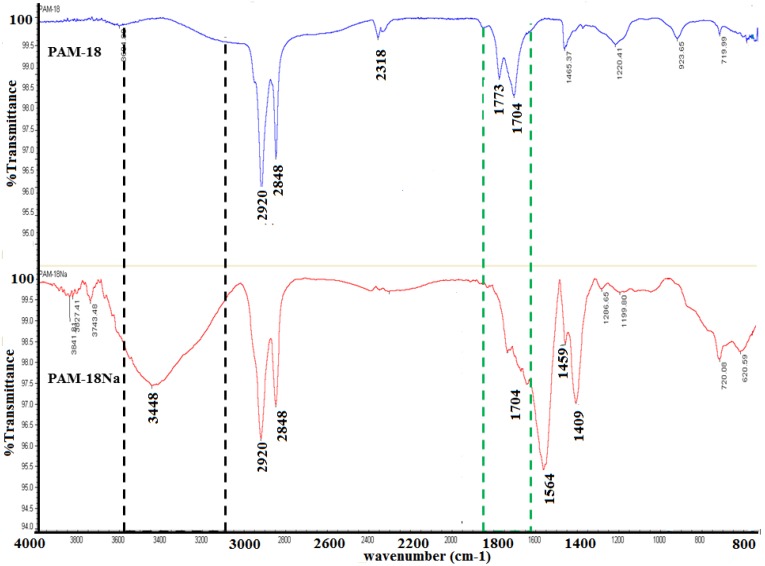
FTIR of PAM-18Na and PAM-18 polymer materials.

The formation of the PAM-4Na and PAM-18Na copolymers was evidenced by a qualitative change in the solubility, changing from a heterogeneous mixture to a completely homogeneous solution. This change is caused by the anhydride group opening in the precursor polymer PAM-4 for PAM-4Na and PAM-18 for PAM-18Na formation, respectively. This led to the formation of two carboxylic acid groups, which were then converted to carboxylates. This transformation was shown by comparison of FTIR spectra between the precursor material and the ionic polymers obtained. The IR spectra showed characteristic signals for both the starting materials (PAM-18, PAM-4) and the polyanion formed (PAM-18Na, PAM-4Na) (Figure 2). Initially, precursors show a signal corresponding to the symmetric and asymmetric stretching of the CH bonds at 2920 and 2848 cm^−1^; also, the signal at 2318 cm^−1^ is observed, representative of the maleic anhydride group. After hydrolysis, the main changes correspond to the disappearance of the signal at 2318 cm^−1^ because of the opening of the maleic anhydride ring and the displacement and variation on the two signals of the carbonyl groups of maleic anhydride, from 1773 and 1704 cm^−1^ to 1704 and 1564 cm^−1^, signals that appear as a result of symmetric stretching of the -C-O-C bonds of the carboxylates formed. Additionally, the presence of polymeric salt is represented by a characteristic asymmetric stretching at the signals of 1459 and 1409 cm^−1^. Finally, the appearance of a band at 3448 cm^−1^ was observed, indicating the presence of a hydroxyl group, from the formation of carboxylic acid groups, suggesting that the ionization process from PAM-18 to PAM-18Na is not full, as expected, and there are some equilibrium processes between carboxyl acid and carboxylate groups, after the maleic anhydride ring opening. In the next figure, the changes mentioned above are presented, using PAM-18 and its respective salt formed, PAM-18Na [15].

### 2.2. Granulometric Properties

The results of the average diameter for powder materials corresponding to trihydrate ampicillin and PAM-4Na, PAM-18Na, PAM-18 and HPMC polymers obtained after the process of manual extrusion were 53, 75, 250 and 53 µm, respectively. Furthermore, certain percentages of compactness by the Carr index showed appropriate values for the study materials, being 10% for ampicillin trihydrate and 7, 15, 17 and 9%, for PAM-4Na, PAM-18Na, PAM-18 and HPMC polymers, respectively. Regarding the value of the repose angle, it was found this value for trihydrate ampicillin was 43°, while for polymeric systems PAM-4Na, PAM-18Na, PAM-18 and HPMC, it was 25, 36, 21 and 29°, respectively. These results indicate low flow ability for ampicillin trihydrate and PAM-18Na polymer, while a good flow ability for the other polymeric materials.

### 2.3. Thermal Characterization of Polymer-Drug Blends

**Figure 3:**
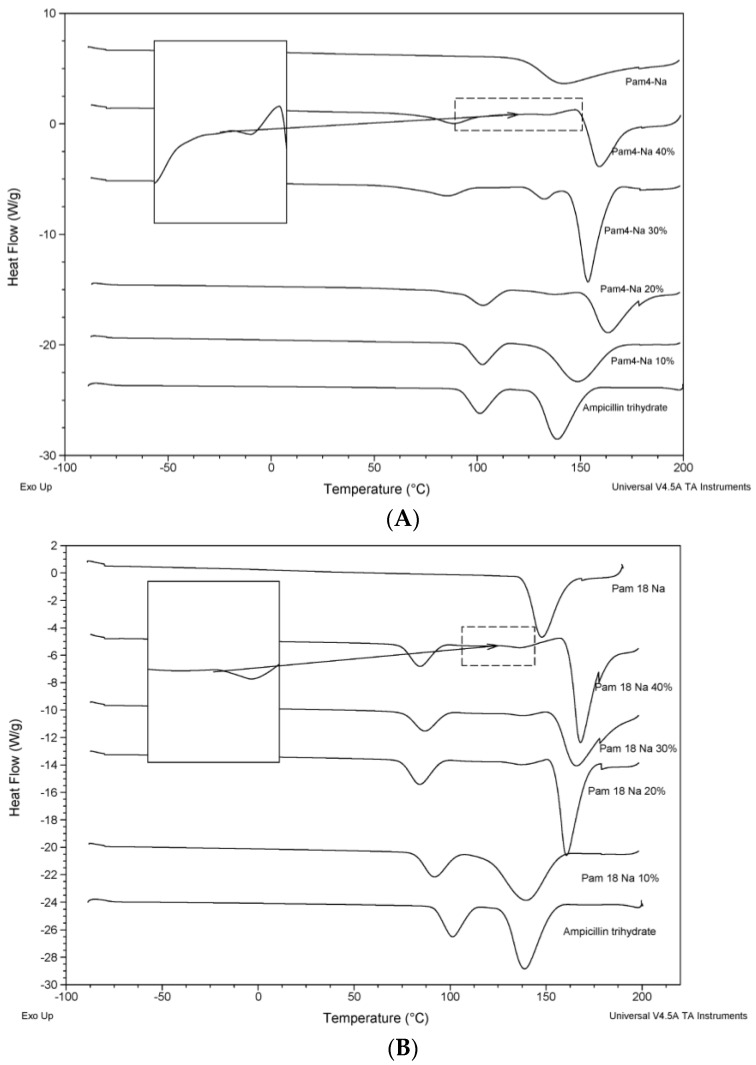

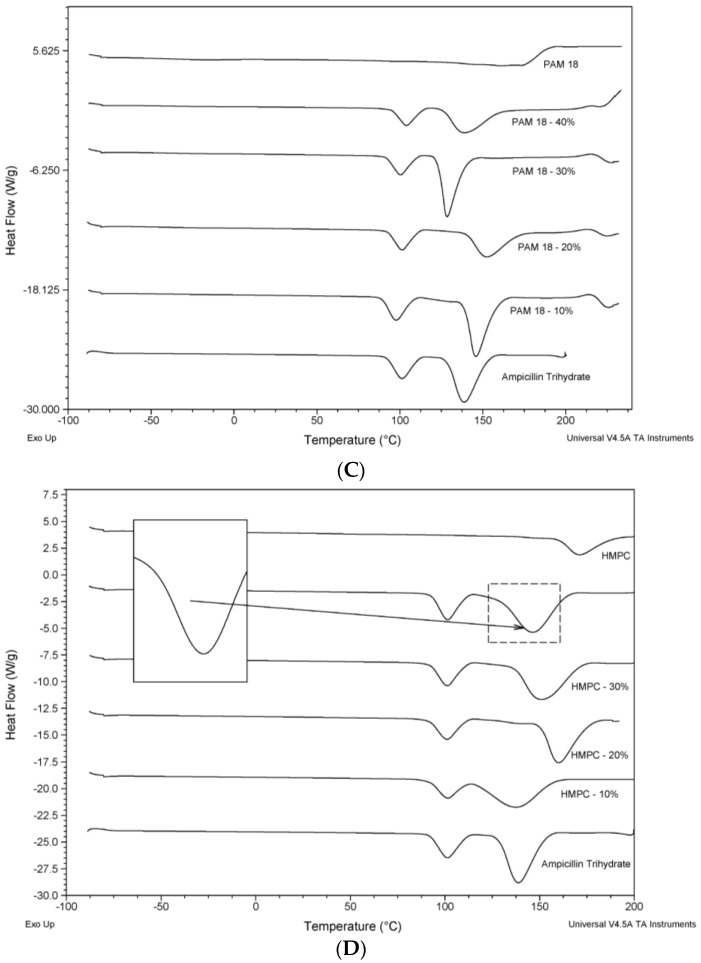
Thermograms of the ampicillin trihydrate blends with the polymeric materials at 10%, 20%, 30% and 40%: (**A**) PAM-4Na; (**B**) PAM-18Na; (**C**) PAM-18 and (**D**) hydroxyl-propyl-methyl-cellulose (HPMC). The endothermic signals are down.

The thermograms for the model drug and the polymeric materials, either alone or in blends, are shown in Figure 3. The thermal characterization analysis indicates the existence of different interaction degrees between polymer materials and the model drug, where the demonstrated behavior is attributed to two phenomena: (1) displacements at temperatures of thermal transition corresponding to pure materials against mixture systems [25]; (2) the formation of a new thermal transition in the blends, as is the case of PAM-18Na and PAM-4Na polymers, which started from 20% (Figure 3A,B). In these cases, an increment of the intensity and thermal energy was observed with the increase in the proportion of polymer in the mixture. These phenomena can be attributed to an interaction between the hydration water of ampicillin trihydrate and the ionic polymer, which generates the signal of water loss (~100 °C) showing displaced transitions at temperatures below the transition of the pure drug, and this means that the interaction is stronger for ionic polymers, PAM-18Na and PAM-4Na, than in the case of neutral polymers, PAM-18 and HPMC. Furthermore, the melt temperature transition for trihydrate ampicillin (~140 °C) is increased when the PAM-18Na and PAM-4Na polymers were added; this change was not observed with the PAM-18 and HPMC polymers (Figure 3C,D). Furthermore, it is remarkable that for each polymer, the curve shows a change in signal of the thermal transition and the formed interactions highlighted before.

### 2.4. Preparation of the Compressed Matrices

The results of the hardness and disintegration time for each tablet of ampicillin trihydrate at different polymer ratios are summarized in the Table 1. It was observed that both the hardness and disintegration time describe different behaviors depending on the polymer proportion inside the tablet [26]. The PAM-4Na polymer shows a tendency to remain constant with values very close to those obtained for the drug alone, while the PAM-18Na polymer reveals an increase in both hardness and disintegration time. The PAM-18 polymer evidences hardness and disintegration time values below those of the drug alone, and they tend to remain constant. The hardness values of HPMC polymer were very similar to those obtained by ampicillin alone, while disintegration values are higher. These results are very interesting because they allow establishing a relationship between the structural characteristics of each polymeric material with the hardness and disintegration properties of the tablet.

The tablets cohesiveness increased using the ionic polymers PAM-4Na and PAM-18Na, while the use of the neutral polymers PAM-18 and HPMC decreased this property. These results exhibit an interesting relationship with the data obtained by DSC, where it was observed that the most significant changes in the thermograms occur in the blends of ampicillin trihydrate with the PAM-4Na and PAM-18Na ionic polymers, indicating that the increase in the tablets hardness is given by specific interactions with the zwitterionic drug forming electrostatic interaction polymers, which have been already described in other works [15]. Furthermore, the data of disintegration time are also very interesting, for the PAM-4Na polymer, which is a hydrophilic low molecular weight material; the matrix disintegration is almost immediate and occurs in a similar way as a conventional release tablet; on the other hand, the PAM-18Na polymer is amphiphilic in nature and shows an increasing disintegration time, due to the high cohesiveness degree of the tablet. This result is quite similar to that obtained in a previous study, where a similar amphiphilic material was used, but with a potassium counter ion [27]. In contrast, the hydrophobic polymer PAM-18 showed a rapid disintegration attributed to the low hardness of the tablet; while the reference polymer HPMC showed higher values of disintegration time because such a matrix might swell in aqueous media forming gel-like structures that are difficult to disintegrate [21]. These results suggest that the mechanisms of disintegration depend on the nature of the polymers and not only the physical interaction, as in the case of thermal analysis. Furthermore, it is important to say that, up to this point the results are just showing an interaction, and this does not mean that the stability of the compressed system is compromised. For related stability, further experiments would be required, and this depends on the type of studies to perform, because the concept is applicable to chemical, physical or thermic stability.

### 2.5. Analysis of the Surface Roughness of the Tablets

**Figure 4:**
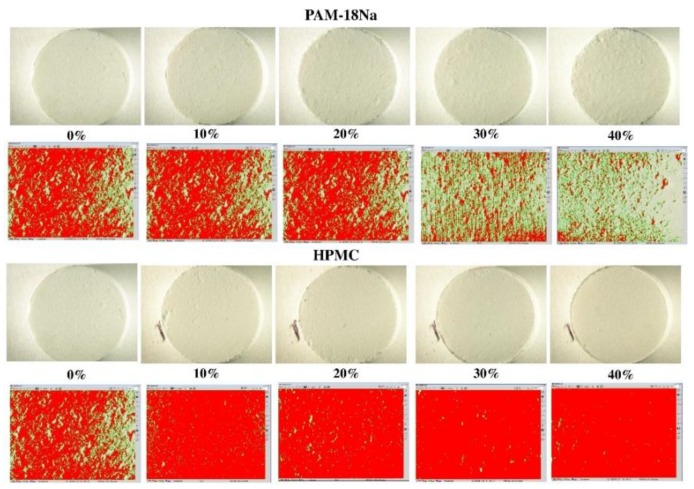
Surfaces of ampicillin trihydrate tablets at different ratios of polymer. The green color indicates a rough surface, while red indicates a smooth surface.

The study of the roughness surfaces using the micro-display method showed that this property depends on the kind and amount of polymer used. Figure 4 shows micrographs and image analysis for tablet surfaces of ampicillin trihydrate with the PAM-18Na and HPMC polymers at different ratios, while the results of the roughness index (*I_R/A_*) are summarized in Table 2.

The tablets with ampicillin alone had an I_R/A_ = 1.18; therefore, its surface tends to be rough. In comparison, the I_R/A_ values of the PAM-4Na polymer increased with the amount of polymer, indicating that the tablets become less rough or smoother. For the polymer PAM-18, it was found that the I_R/A_ tends to remain constant with the percentage of polymer and the I_R/A_ values indicating that the surface is rough, while HPMC polymer displayed a similar behavior to that described for the PAM-4Na polymer, wherein the surface gradually becomes smoother. This effect has already been described for such polymers, which tend to form plastic films with similar properties [28]. Therefore, the surfaces of ampicillin trihydrate tablets tend to become rougher with those polymers containing alkyl chains of greater length in their structures, such as PAM-18Na and PAM-18, than with those having short alkyl chains, as PAM-4Na and HPMC.

### 2.6. Contact Angle Measurements (θ_c_)

The contact angle measurements are related to the proportion of polymer within the ampicillin trihydrate tablets; it reveals that contact angle and polymer percentages have a proportional relationship, suggesting that the tablet surface tends to become more hydrophobic with the increasing of polymer in the system [29,30,31]. The results of the variation of *θ_c_* using different reference liquids in addition to the physical properties of these are summarized in Table 3. In the case where ultrapure water was used as the liquid reference, it is necessary to consider that for each analysis of *θ_c_*, the largest contribution to the total surface tension (*γ_total_*) corresponds to the polar surface tension (γ^P^), and this represents approximately 70%. Thus, each analysis is set in terms of polar attractive or repulsive hydrophobic interactions. Here, in the case of ampicillin alone with water as the reference liquid, *θ_c_* is 61.52°, suggesting the spreading and wetting of the water droplet on the tablet surface. On the other hand, for tablets containing PAM-4Na, it is observed that increasing the amount of polymer leads to a slight and gradual increase of *θ_c_*. In the case when the tablets contain PAM-18Na, the results are very close to those previously found with a similar polymer, such as PAM-18K [27], where at polymeric proportions of 10% and 20%, *θ_c_* is much lower compared to the value shown by the surfaces of the ampicillin tablets, whereas at polymer proportions of 30% and 40%, *θ_c_* values are greater and very close to 90°, suggesting a transition from a hydrophilic to a hydrophobic surface.

Moreover, *θ_c_* values for PAM-18 and HPMC polymers also show that slight increases exist with the proportion of polymer within the tablet, but with closer values at 90°. Therefore, *θ_c_* variation according to the percentage of polymer in tablets and using ultrapure water as the reference liquid can be explained based on the chemical structure of the system components. For ampicillin trihydrate, there are a variety of polar functional groups that allow it to interact attractively with the ultrapure water droplet leading to the spreading phenomenon. For the polymer PAM-18Na, it is possible that a specific orientation of the comonomeric groups on the surface tablets with respect to the polymer occurs, where at low proportions (10% and 20%), the alkyl chains locate inwardly, leaving the carboxylate groups out of the surface, turning it more hydrophilic, while at higher proportions (30% and 40%), the effect is the opposite, and the surface becomes hydrophobic, as previously described for the polymer PAM-18K [27]. For the polymer PAM-4Na, the alkyl chain length is not large enough to generate a hydrophobic repulsion as marked as with the polymer PAM-18Na; thus, the *θ_c_* observed is below 90°, indicating that the hydrophobicity degree of the tablet surface does not vary considerably [34,35,36]. In the case of PAM-18 polymer, *θ_c_* values are very close to 90°, and there is no variation in the amount of these with polymer amount, suggesting that the tablet surface remains hydrophobic. The HPMC polymer has a similar behavior as PAM-18, where the values obtained are close in magnitude and also an increase in the hydrophobicity surface degree exist according to the polymer proportion.

Regarding *θ_c_* values obtained using ethylene glycol and isopropanol as reference liquids, it was observed that there are no marked tendencies related to the polymer amount within the tablet. However, *θ_c_* values are lower than those obtained with water as the test liquid. These results can be explained by considering the dielectric constant values (ε), the polar (*γ^P^*) and dispersive (*γ^d^*) surface tensions for the two organic liquids of reference. The ethylene glycol is a high polarity liquid (ε = 68), and *θ_c_* values are in a range between 48°and 70°; this is slightly lower than those obtained with water in all cases. The apolar solvent isopropanol (ε = 17.9) shows decreased *θ_c_* values ranging between 13° and 22°. These results indicate that the liquid spreading on the tablet surface is thermodynamically favored as the polarity decreases.

### 2.7. Determination of W_adh_ and SFE

The results of the work of adhesion (*W_adh_*) and surface free energy (*SFE*) for ampicillin tablets with different polymeric materials are summarized in Table 4.

Regarding the *W_adh_* values obtained by the Young–Dupré model and using ultrapure water as the reference liquid, it was found that these depend on the type and amount of polymer within the tablet. For the PAM-4Na polymer, a slight decrease is observed in W*_adh_*, when the amount of polymer is elevated, indicating that the interactions on the tablet surface are attractive and hydrophilic in nature. The polymer PAM-18Na shows a marked change in W*_adh_* related to the polymer proportion, from 109.9 down to 64.8 mJ/m^2^. This result is in agreement to that previously found for a similar system [27], which suggests a change in the type of interactions between the liquid of the reference and the tablet surface, where a low percentage of polymer (10%–20%) contributes to hydrophilic interactions, while higher percentages (20%–40%) lead to hydrophobic interactions. For PAM-18 and HPMC polymers, it was found that *W_adh_* is practically constant with values between 79 and 90 mJ/m^2^, suggesting that the interaction in the interface area is hydrophobic, which is indifferent to the polymer proportion.

Furthermore, the values obtained from *SFE_tota_*_l_ using the OWRK model indicate that a very good fit to the semi-empirical model exists, and thus, it is possible to establish the dispersive and polar contributions of SFE present in ampicillin tablets. To achieve a better assessment of this, the polar/dispersive index (I_p/d_) was defined as the ratio between the *SFE^p^* and *SFE^d^* and thereby a balance between the attractive interactions of the polar type (dipole-dipole and bridge set hydrogen) and dispersive interaction (London interactions) present in the surface region. Thus, values of I_p/d_ ≤ 1.0 indicate a prevalence of London-type dispersive interactions, while values ≥ 1.0 indicate a prevalence of polar interactions, like hydrogen bonds or dipole-dipole. On the other hand, a value of 1.0 indicates that both interactions are present in the same proportion. 

In relation to the surfaces formed by ampicillin trihydrate alone, it has a value of I_p/d_ = 6.4; this means that the interactions that dominate the tablet surface are of the polar type. For the PAM-4Na polymer, it was observed that the values of *I_p/d_* are greater than 1.0 in each of the cases evaluated, indicating that the interactions are also of the polar type. In the case of the PAM-18Na polymer, it was determined that I_p/d_ values depend on the percentage of polymer in the tablet, with values of 6.2 and 20.2, for minor amounts (10% and 20%), and 0.8 and 0.1, for higher amounts (30%–40%); suggesting that there is a transition between the two types of attractive interactions in the surface, where at low rates, the dipole-dipole polar interactions prevail, while at high proportions, the London dispersion interactions predominate. A very interesting result is observed for the PAM-18 polymer. At first, it seems contradictory, since, being a hydrophobic material, one would expect that the greatest contribution to SFE were dispersive, and thus, the values I_p/d_ should be less than one. However, the result is the opposite; we consider that this is produced because the interactions with ampicillin are located in the surface area rather than with the polymer itself, since such tablets showed a very low cohesiveness and a tendency to generate non-homogeneous mixtures. The I_p/d_ values obtained for HPMC tend to remain close to one in most cases, suggesting that in the surface of the tablets, the two types of interactions predominate, whereas at 40% of polymer, the London dispersive interactions prevail. All of these results are consistent with those obtained previously.

### 2.8. Variation of Contact Angle vs. Time

**Figure 5:**
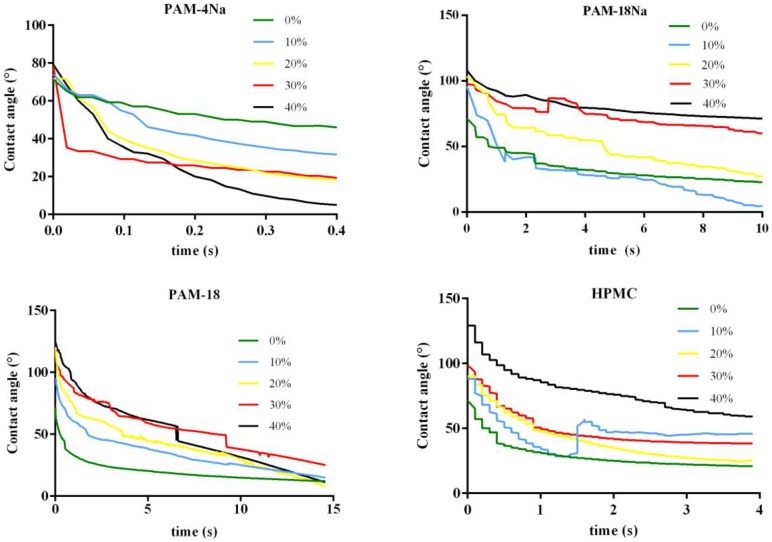
Profiles of *θ_c_* vs. *t* for ampicillin trihydrate tablets alone and with polymeric materials at different rates. Time values correspond to the normalized values of drop age.

In the case of uneven and rough surfaces, it is very important to know the rate of liquid penetration into the tablets, since this phenomenon can influence the release of a particular drug in media dissolution. This absorption rate can be parameterized evaluating variations of *θ_c_* for the drop age, which corresponds to the time that it takes for a liquid drop to disappear from the moment of its fall on the surface. Thus, the AUC profiles values from *θ_c_* vs. *t* are equivalent to the water absorption rate by a specific surface. The time used for AUC calculations is different for each polymeric material because of its characteristics and the different water absorption rates; this corresponds to the last time points in each *x*-axis. Figure 5 shows the contact angle with respect to the time profiles (*θ_c_* vs. *t*) for ampicillin trihydrate tablets, while the results of the AUC for such profiles are summarized in Table 5.

The results show that a strong dependence of AUC regarding the type and amount of polymer present in each tablet exists. The AUC values for the PAM-4Na polymer tended to remain constant with the polymer amount, besides being the lowest in magnitude compared to the other polymers used in the study, suggesting that the water absorption rate is very fast with this polymer. This result is very consistent when related with contact angles or previous surface free energy results (Table 3 and Table 4), where it was found that the nature of these surfaces is quite polar, and thus, the phenomenon of relaxation with water is highly favored thermodynamically. A transient behavior was found for the PAM-18Na polymer, where low percentages of this (0%–10%) are observed at minimum values of AUC, while high percentages (30%–40%) for the highest AUC values. This result suggests that the water absorption rate is faster at low polymer proportions and is slower at higher amounts, being very consistent with those previously obtained for *θ_c_* (Table 3), where it was found that these surfaces become more hydrophobic with the increase of the polymer. Furthermore, the PAM-18 and HPMC polymers show a gradual increase between AUC and the polymer percent, suggesting that the water absorption rate is dependent on the polymer amount in the tablets, which is also consistent with that described in the previous results.

### 2.9. In Vitro Dissolution Tests of the Model Drug

**Figure 6:**
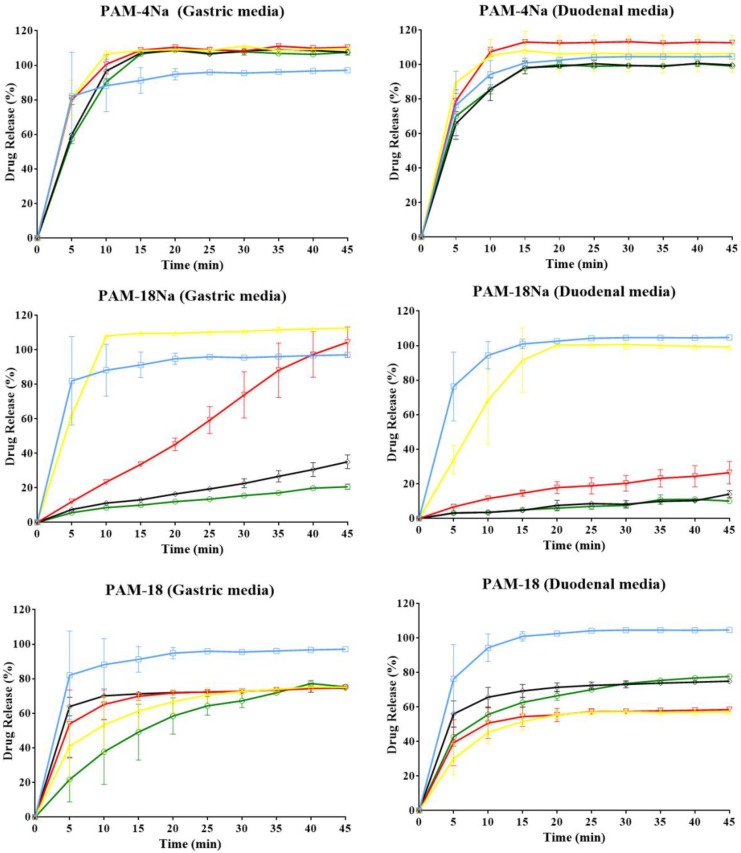

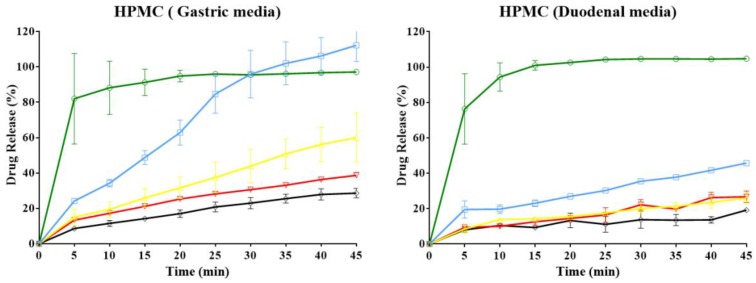
In vitro dissolution profiles of ampicillin trihydrate tablets using different polymer ratios: ◯ = 0%, ☐ = 10%, △ = 20%, ▽ = 30% and ◊ = 40% and two media for physiological simulation.

The results of the in vitro dissolution profiles for each ampicillin trihydrate tablet with different polymer ratios using two simulation physiological media, such as gastric (buffer solution pH 1.2; 0.15 M) and duodenal media (buffer solution pH 7.4; 0.15 M), are presented in Figure 6. Here, it was found that there is a strong dependence of the model drug release profiles on the dissolution media, the type and the polymer proportion within the tablet. The dissolution profiles of the PAM-4Na polymer, at all percentages of polymer evaluated in both media, have a behavior similar to an immediate or conventional release dosage form; this means that 85% of drug release is reached in the first 15 min of the dissolution test [37,38]. This result is consistent with previous observations, where it was described that the tablet surface is highly polar and has a high water absorption rate, thus favoring free diffusion of the drug into the bulk solution regardless of the polymer concentration in the matrix and the media pH [39]. Similar behaviors were observed for the dissolution profiles of PAM-18Na and HPMC polymers, where the increase of the polymer proportion modulates the dissolution profile changing from a conventional to a modified release form [40].

The dissolution profiles of the PAM-18 polymer change as the polymer amount increases; however, the changes are not as marked as with PAM-18Na and HPMC; this can be explained by the low hardness of the tablets containing the PAM-18 polymer. There is a faster disintegration of tablets, leaving the drug more exposed to the environment, so that the diffusion-dissolution process occurs [41,42]. In order to compare parametrically each of these profiles, the values of dissolution efficiency (*DE*) were obtained for each tablet in both dissolution media. These results are summarized in Table 6.

The results of *DE* describe the same behavior discussed above; however, some differences have been observed between the dissolution medium, the type and the polymer proportion within the tablet. In the case of the PAM-4Na polymer, it was found that *DE* is higher when the percentages of polymer are between 10% and 20% than when ampicillin is alone, while between 30% and 40% of polymer, a slight decrease occurs regardless of the dissolution media. In the case of PAM-18Na and HPMC polymers, it was observed that the *DE* of both decreases significantly with increasing polymer amounts in the tablet, the effect being more marked in the gastric simulation media than in the duodenal. Moreover, it was observed that with 10% PAM-18Na polymer, the dissolution efficiency is around 98%, similar to that described by conventional delivery systems [37], whereas for HPMC at 10%, a modular release occurs. Therefore, the PAM-18Na polymer needs higher concentrations to control the release compared to the reference material, HPMC. Furthermore, it was seen in both cases that *DE* is higher in the gastric media than in the duodenal solution, where this class of polymers is neutralized and is likely to form gel-like states, which control the release mechanism, leading to slower dissolution rates [43]. The PAM-18 polymer displays a constant *DE* and values lower than 70% in both dissolution media, which seems contradictory if *θ_c_* behavior relative to the polymer amount in the tablet is analyzed; this showed that the surface becomes more hydrophobic, and thus, the drug release should be controlled. However, the results of the hardness and disintegration analysis showed that these matrix tablets are very erodible, where the drug can diffuse faster into the bulk dissolution, as observed with PAM-4Na. Finally, we can say that in general, the results of *DE* are very consistent with those previously found for the hardness and disintegration of tablets, the I_R/A_ of the surfaces, the thermodynamic properties *θ_c_*, *W_adh_* and SFE and, also, the rate of water absorption. 

### 2.10. Kinetic Study of Drug Release

In order to explain further the dissolution profiles found in terms of the possible mechanisms of drug release from the matrix systems, a kinetic analysis was performed using several semi-empirical models. The results are summarized in Table 7. For the PAM-4Na polymer, it was observed that in both dissolution media, the data fit the Higuchi model [41], suggesting that the release mechanism is controlled purely by drug diffusion from the matrix compressed to the bulk of the solution and does not depend on the polymer (Fickian diffusion). This result is consistent with what was observed in the dissolution profiles, showing a typical conventional release process. In the case of PAM-18Na, very similar results were observed to those obtained previously with the analogous PAM-18K [27], where it was found that depending on the percentage of polymer and the dissolution media, the data adjusted to different models. In the case of 10% of polymer in gastric media and 10% and 20% in duodenal media, data fit well to the Higuchi model, suggesting that drug release is given by the Fickian diffusion process; while for the percentages of polymer between 20% and 40% in gastric media and 30% and 40% in duodenal media, data fit well the Korsmeyer–Peppas model, with different values of *n*. For the duodenal media, values of *n* ≈ 1 are observed, suggesting that the release mechanism is anomalous and that this is controlled by the relaxation of the polymer chains [36,37], through a process where the dissolution medium penetrates the compressed matrix forming pores and then erodes it; while for the gastric media, values of *n* > 1 are observed, suggesting that the release mechanism is super Case II of transport, wherein the polymeric matrix makes a transition from a glassy state to a relaxed rubber state [42,44]. The dissolution data of the PAM-18 polymer do not adjust to the models used, because the matrix is highly porous, less compact and erodible, and therefore, it is necessary to evaluate other types of kinetic models, such as Hopfenberg [37] and Hixson–Crowell [45], which are the most used for this type of matrix. Finally, in the case of HPMC, which corresponds to a model material of controlled release, it was observed that the gastric environment data fit well to the Korsmeyer–Peppas model with *n* values between 0.5 and one, suggesting an anomalous diffusion mechanism, while in the duodenal environment, data fit best to a model of order-one, which is typical for the release of polar drugs from porous matrices as used in this study.

## 3. Materials and Methods

### 3.1. Materials

The polymeric precursors and reference materials were: poly(maleic anhydride-alt-octadecene) or PAM-18 with M_W_ of 30,000–50,000 (Sigma-Aldrich, St. Louis, MO, USA), poly(isobutylene-*alt*-maleic anhydride) or PAM-4 with M_W_ ~6000 (Sigma-Aldrich, St. Louis, MO, USA) and HPMC (Sigma-Aldrich, St. Louis, MO, USA). The model drug was ampicillin trihydrate (Fersinsa Gb), which was provided by Tecnoquimicas Laboratories S.A (Cali, Colombia) and was used as received. The reagents used for the preparation of the dissolution media were: KOH, KCl, KH_2_PO_4_ and K_2_HPO_4_ from Merck (KGaA, Darmstadt, Germany), used as received. Moreover, KCl was used to adjust ionic strength. Type II water obtained from a purification system (Millipore Elix essential, Merck KGaA, Darmstadt, Germany) was used to prepare all of the buffer solutions. For measurements of contact angle, the following liquids were used as a reference: Type I water obtained from a purification system (Arium pro Sartorius Stedim biotechnology VF, Göttingen, Germany), isopropanol (LiChrosolv, Merck KGaA, Darmstadt, Germany) and ethylene glycol (Merck KGaA, Darmstadt, Germany).

### 3.2. Obtaining and Characterization of Polymers

The PAM-18Na polymer was obtained and characterized according to previously-described methods [15,27]. To do this, the PAM-18 was reacted with an equimolar amount of NaOH. The modification was carried out at room temperature for one hour with moderate agitation. Subsequently, the polymer solution was dialyzed using a 12-kD cut-off cellulose membrane (Sigma-Aldrich, St. Louis, MO, USA) until a constant conductivity value of about 5 μS/cm was reached. The PAM-4Na solution was not dialyzed due to its low molecular weight. Each polymer solution was lyophilized in an Eyela freezer (FDU Model 1110, Eyela, Tokyo, Japan) to obtain powder materials, which were subsequently extruded manually with a mesh of 75 microns. The structural characterization of the polymeric material was carried out by FTIR spectroscopy (Nicolet 6700, Thermo Fisher Scientific, Waltham, MA, USA).

### 3.3. Methods

#### 3.3.1. Granulometric Properties

The granular diameter was determined for each powder material by tapping using a Ro-Tap RX-29 screening system (wsTyler, Mentor, OH, USA); the percentage of compressibility was determined from Carr’s index, using a density meter (Logan Tap-2S). The flow degree was obtained by determining the angle of repose using a fixed funnel method.

#### 3.3.2. Thermal Characterization of Polymer-Drug Blends

The model drug, the polymeric materials and their respective blends in proportions of 10%, 20%, 30% and 40% (*w*/*w*) were analyzed in a Q2000 differential scanning calorimeter (DSC; TA Instruments, New Castle, DE, USA) calibrated with indium T_f_ = 155.78 °C ∆H_f_ = 28.71 J/g. DSC analysis was carried out using three heating-cooling cycles from −90 °C (183.15 K) to 200 °C (523.15 K) with a heating rate of 20 °C/min.

#### 3.3.3. Preparation of the Compressed Matrices

The tablets were made using a homemade tablet press with 1/4 inch in diameter flat stainless steel punches. For each tablet, 500 mg of ampicillin trihydrate in different proportions mixed with the polymers, corresponding to 0%, 10%, 20%, 30% and 40% *w*/*w*, were used. A compression pressure of 300 psi was applied for 10 s in each tableting. The hardness was determined using a durometer (Logan HDT-400), while the disintegration time was determined by an automated disintegrator (Logan USP DST-3) in Type II water at 37 °C.

#### 3.3.4. Analysis of Surface Roughness of the Tablets

Determining the roughness degree for each tablet was carried out by the micro-display high magnification technique using a micro-stereoscope (Nikon SMZ1500, Nikon Industries Inc., Melville, NY, USA). The “surface roughness” was estimated with the NIS-Elements Advanced Research software (Nikon Industries Inc., Melville, NY, USA). For this, several images of each tablet were captured and used to analyze the contrast of pixels in light and dark areas under the following conditions: region of interest (ROI) 189 × 120 pixels, binary threshold, function intensity (left = 90), (right 200) and 0.75× optic zoom. All tests were performed under homogeneous conditions of incident light intensity, temperature and relative humidity. Finally, the relative roughness index (I_R/A_) indicates the surface roughness of the tablets, and it is defined as:

(1) $$I_{R/A}=\frac{\left(\frac{ANR}{R}\right)}{ANR}=\frac{1}{R}$$

where *ANR* is the not roughened area of the image and *R* is the roughness factor, both parameters given by the software. When I_R/A_ ≤ 1.20, it is established that the surface tends to be rough, while *I_R/A_* ≥ 1.30 suggests that the surface is smooth. Furthermore, values between 1.20 and 1.30 set an intermediate state between a smooth and rough surface.

#### 3.3.5. Contact Angle Measurements

Determination of the static contact angle was carried out on the surfaces of each ampicillin trihydrate tablet with the polymeric system, immediately after being manufactured. The sessile drop method was used by a contact angle meter (OCA15EC Dataphysics Instruments, Filderstadt, Germany) with a software driver (Version 4.5.14 SCA20). Data capture was recorded on an imaging development system IDS video camera, where the information from a range between 400 and 800 frames was taken as a reference point as a static angle. Moreover, the point capture of contact angle was defined as the reflected light of incident drop completely disappearing (about 1 s, since leaving the dispensing system). Drop volumes were in a range of 5 × 10^−3^–15 × 10^−3^ mL, and the liquid deposition fall was fixed to 1 cm for all assays. Each measurements were carried out at 22 ± 1 °C and 60% ± 5% of relative humidity. The contact angle was measured at least three times on different sites of the tablets surface. Each datum reported is the average of triplicate measurements; however, it was not possible to establish the hysteresis for this parameter due to the deformation of the surface tablets given by the absorption of the reference liquids.

#### 3.3.6. Determination of *W_adh_* and *SFE*

The work of adhesion (*W_adh_*) was determined for each surface of the ampicillin trihydrate tablets with different polymeric materials using the Young–Dupré model [46] and surface free energy (SFE) from the OWRK model [47]. Ultrapure water was used as reference liquid to determine *W_adh_*, while in the OWRK model, propanol, ethylene glycol and water were used.

#### 3.3.7. Contact Angle vs. Time

The contact angle change in the (*θ_c_*) as a function of elapsed time or drop age on the solid surface allows one to establish in an indirect way the water absorption rates on the compressed tablet surface. This measurement was carried out by the non-static contact angle tracking function provided by Dataphysics software. The data were taken from software as contact angle (*θ_c_*) vs. drop age (s); later, the area under the curve (AUC) was plotted against the percentage of polymer added, using GraphPad Prism 6 software. For the test, ampicillin trihydrate tablets with different proportions of polymer materials, PAM-4Na, PAM-18Na, PAM-18 and HPMC, corresponding to 0%, 10%, 20%, 30% and 40% *w*/*w*, were used. Every test was carried out until the point where the instrument failed to register more values of contact angle variation, either because all of the liquid was absorbed or because a deformation took place on the solid surface. 

#### 3.3.8. In Vitro Dissolution Tests

Chemical stability assays for the ampicillin trihydrate under the study conditions were performed due to the degradability of the beta-lactamic ring with respect to temperature and pH of the media [48]. For this, a stress stability test was carried out for 6 h at 37 °C. The ampicillin solutions were prepared in three different dissolution media (ultra-pure water and buffer solution pH 7.4 and pH 1.2). Consecutive samples were taken every 10 min and analyzed by HPLC with an ultra-array detector (Hitachi, VWR) at 254 nm and 30 °C. For this, a mixture of acetonitrile and phosphate buffer with a 3:7 ratio (pH: 5.5) as the mobile phase and a column Nova-pack C18 3.9 × 75 mm as the stationary phase were used. Finally, the kinetic integral method was used, where the zero-, one- and two-order models were evaluated. The stability study results established that the maximum duration of the assay should not be longer than 1 h.

The dissolution test was carried out using the paddle method on a previously calibrated tester (apparatus II, Vision G2 Classic 6-Hanson, Chatsworth, CA, USA). The paddle speed was 100 rpm at a temperature of 37.0 °C ± 0.5 °C. The media volume of simulated gastric and plasma conditions (buffers pH 1.2 and pH 7.4 with ionic strength of 0.15 M, respectively) was 900 mL. Each dissolution test was carried out for 45 min, where a 5-mL sample was taken with replacement at predetermined time intervals. The samples were filtered through a 0.45-μm filter. Determination of the ampicillin amount in ultra-pure water and buffer solution pH 7.4 was carried out by UV spectrophotometry at 256 nm at 37.0 °C (310.15 K) using a UV spectrophotometer (Shimadzu, Kyoto, Japan) coupled to a temperature control system. The data obtained from the in vitro dissolution profiles are reported as the average dissolution efficiency (DE) of the tablet [27,37,49]. Finally, the kinetic models of order-zero [45], order-one [49], Higuchi [50,51] and Korsmeyer–Peppas [36,37] were evaluated to describe the release mechanism of the model drug in the different compressed matrices.

### 3.4. Data Processing and Analysis

The data were tabulated and analyzed using Microsoft Excel and GraphPad Prism 6, respectively. The homogeneity of variance in the data was analyzed using Bartlett’s test. Statistical comparisons were made using a one-way ANOVA. The Bonferroni post-hoc test was used to determine significant differences between the two independent groups. A confidence level of 95% was adopted. Data are expressed as the mean ± standard deviation.

## 4. Conclusions

The physical, surface and kinetic characteristics of ampicillin trihydrate matrix tablets are strongly affected by the type and amount of polymer used. In the case of the hardness and disintegration time, these are most affected when the polymers used are ionic in nature, such as PAM-4Na and PAM-18Na, where greater interaction effects exist in the solid state, than when the polymers are neutral and hydrophobic, as PAM-18, where the hardness and disintegration times are very low. Furthermore, the surfaces of the tablets tend to become rough and hydrophobic with increasing amounts of those polymers that contain longer alkyl chain lengths in their structure, such as PAM and PAM-18Na. Furthermore, the water absorption rate depends on the degree of polymer hydrophobicity, being faster when the polymers with short alkyl chains (PAM-4Na and HPMC) are used than with those of long alkyl chains (PAM-18Na and PAM-18). Finally, the release profiles depend on the type and amount of polymer used. Thus, PAM-4Na is not able to control the release of the model drug, while HPMC, depending on the proportion in the tablet, modulates the release through an anomalous diffusion mechanism. In the case of the PAM-18Na polymer, it shows that the release mechanism depends on the amount of polymer going from a Fickian release at low rates to a controlled relaxation of polymer chains to release greater proportions.

## Figures and Tables

**Table 1:** Results of hardness and disintegration time for the tablets of ampicillin trihydrate at different polymer ratios.

Polymer	% Polymer	Tablets Hardness (kp)	Disintegration Time (min:s ± s) at 37 °C
PAM-4Na	0	8.65 ± 0.33	4:30 ± 0
	10	7.42 ± 0.38	4:03 ± 5
	20	10.04 ± 0.53	5:00 ± 0
	30	9.84 ± 1.64	5:50 ± 0
	40	12.69 ± 0.76	5:50 ± 0
PAM-18Na	0	8.65 ± 0.33	4:48 ± 2
	10	10.37 ± 0.22	4:30 ± 0
	20	12.17 ± 1.09	9:35 ± 1
	30	13.73 ± 0.52	23:14 ± 9
	40	16.80 ± 0.19	32:48 ± 5
PAM-18	0	8.65 ± 0.33	4:30 ± 0
	10	2.91 ± 0.59	2:13 ± 2
	20	2.92 ± 0.25	1:34 ± 3
	30	2.25 ± 0.44	1:12 ± 2
	40	2.73 ± 0.15	1:42 ± 1
HPMC	0	8.65 ± 0.33	4:30 ± 0
	10	6.66 ± 0.47	>4 h
	20	7.81 ± 0.66	>4 h
	30	8.16 ± 0.97	>4 h
	40	8.73 ± 0.41	>4 h

**Table 2:** Roughness index (I_R/A_) of ampicillin trihydrate tablets at different polymer proportions.

% Polymer	I_R/A_			
	PAM-4Na	PAM-18Na	PAM-18	HPMC
0%	1.18	1.18	1.18	1.18
10%	1.10	1.17	1.14	1.12
20%	1.21	1.17	1.20	1.39
30%	1.33	1.10	1.12	1.37
40%	1.49	1.08	1.13	1.54

**Table 3:** Contact angle values (*θ_c_*) for ampicillin trihydrate tablets at different ratios of polymers using several reference liquids.

Polymer	% Polymer	Contact Angle (°)		
		Water	Ethylene Glycol	Isopropanol
PAM-4Na	0	61.5 ± 2.3	61.4 ± 4.2	16.8 ± 1.6
	10	64.8 ± 4.9	66.6 ± 2.3	20.2 ± 2.9
	20	68.8 ± 4.2	57.7 ± 1.6	15.2 ± 0.4
	30	69.8 ± 5.1	48.2 ± 2.0	12.9 ± 0.9
	40	71.6 ± 0.6	69.8 ± 7.4	13.4 ± 0.8
PAM-18Na	0	61.5 ± 2.2	61.4 ± 4.2	16.8 ± 1.6
	10	58.9 ± 2.9	52.7 ± 2.3	18.2 ± 3.3
	20	45.3 ± 2.1	50.8 ± 2.5	21.0 ± 3.7
	30	83.2 ± 3.1	64.2 ± 1.1	20.6 ± 2.6
	40	95.6 ± 2.5	60.8 ± 2.7	16.0 ± 2.5
PAM-18	0	61.5 ± 2.3	61.4 ± 4.2	16.8 ± 1.6
	10	83.6 ± 2.5	70.0 ± 2.4	19.3 ± 1.8
	20	78.9 ± 1.4	68.8 ± 0.8	17.3 ± 0.4
	30	79.9 ± 4.0	69.1 ±3.5	18.4 ± 3.6
	40	79.0 ± 0.5	67.6 ± 3.4	15.3 ± 2.7
HPMC	0	61.5 ± 2.3	61.4 ± 4.2	16.8 ± 1.6
	10	73.5 ± 4.0	57.2 ± 2.5	19.6 ± 4.6
	20	77.9 ± 3.2	54.8 ± 1.7	16.3 ± 2.0
	30	76.3 ± 2.4	57.9 ± 1.4	20.4 ± 1.2
	40	84.2 ± 3.4	61.4 ± 2.8	21.6 ± 3.7

Liquid physical property: (1) water (*γ_total_* = 72.1 mN/m, *γ^D^* = 19.9 mN/m, *γ^P^* = 52.2 mN/m and ε = 80.1); (2) ethylene glycol (*γ_total_* = 48.0 mN/m, *γ^D^* = 29.0 mN/m, *γ^P^* = 19.0 mN/m and ε = 68); and (3) isopropanol (*γ_total_* = 23.0 mN/m, *γ^D^* = 19.5 mN/m, *γ^P^* = 3.5 mN/m and ε = 17.9). Values taken from Birdi, K.S. [32], Ohm, A. and Lippold, B.C. [33].

**Table 4:** Values of *W_adh_* and *SFE_total_* for ampicillin trihydrate tablets alone and with polymeric materials at different proportions.

Polymer	% Polymer	*W_adh_* (mJ/m^2^)	Surface Free Energy (mJ/m^2^)			(*R*^2^)	(s)	I_p/d_
			*SFE_total_*	*SFE^d^*	*SFE^p^*			
PAM-4Na	0%	106.1 ± 2.7	39.5 ± 2.5	5.3 ± 0.8	34.2 ± 3.4	0.983	4.0	6.4
	10%	102.3 ± 5.9	36.3 ± 5.4	5.2 ± 0.9	31.1 ± 6.3	0.982	4.3	5.9
	20%	97.7 ± 5.3	33.3 ± 3.7	8.7 ± 1.6	24.6 ± 5.3	0.999	0.8	2.8
	30%	96.6 ± 6.6	33.6 ± 3.6	11.2 ± 2.8	22.4 ± 6.4	0.993	2.0	2.0
	40%	94.5 ± 0.3	30.1 ± 1.6	7.4 ± 1.6	22.7 ± 1.0	0.972	4.1	3.0
PAM-18Na	0%	106.1 ± 2.7	39.5 ± 2.5	5.3 ± 0.8	34.2 ± 3.4	0.983	4.0	6.4
	10%	108.9 ± 1.8	42.3 ± 1.8	5.8 ± 0.7	36.5 ± 2.6	0.997	0.8	6.2
	20%	122.4 ± 1.7	57.5 ± 1.7	2.7 ± 0.1	54.8 ± 1.7	0.986	1.9	20.2
	30%	80.3 ± 4.0	24.6 ± 0.9	13.4 ± 1.6	11.1 ± 2.6	0.989	1.4	0.8
	40%	64.8 ± 1.3	25.4 ± 1.1	22.6 ± 1.6	2.8 ± 0.5	0.973	1.7	0.1
PAM-18	0%	106.1 ± 2.7	39.5 ± 2.5	5.3 ± 0.8	34.2 ± 3.4	0.983	4.0	6.4
	10%	79.9 ± 2.0	23.4 ± 0.1	12.2 ± 1.5	11.2 ± 1.6	0.992	1.6	0.9
	20%	85.7 ± 1.6	25.5 ± 0.7	10.3 ± 0.4	15.2 ± 1.2	0.986	2.3	1.5
	30%	84.3 ± 4.9	25.0 ± 2.3	10.7 ± 1.0	14.3 ± 3.4	0.990	2.0	1.3
	40%	85.6 ± 0.5	25.7 ± 0.6	10.9 ± 1.0	14.8 ± 0.5	0.992	1.9	1.4
HPMC	0%	106.2 ± 2.8	39.5 ± 2.5	5.3 ± 0.8	34.2 ± 3.4	0.983	4.0	6.4
	10%	92.2 ± 4.8	30.1 ± 2.2	10.4 ± 2.5	19.7 ± 4.6	0.998	0.7	1.9
	20%	86.9 ± 3.8	28.1 ±1.1	13.4 ± 2.0	14.7 ± 2.3	0.992	1.9	1.1
	30%	88.9 ± 2.9	28.3 ± 1.4	11.4 ± 0.9	16.9 ± 2.4	0.999	0.8	1.5
	40%	79.2 ± 4.3	24.4 ± 1.1	15.2 ± 1.9	9.2 ± 3.1	0.990	1.0	0.6

*SFE^d^* and *SFE^p^* correspond to dispersive and polar contribution in *SFE_total_*. *R*^2^ and s are the linear determination coefficient and standard deviation for *SFE_total_* by the OWRK model, while I_p/d_ is the polar/dispersive index.

**Table 5:** AUC values of *θ_c_* vs. *t* profiles for ampicillin trihydrate tablets at different ratios of polymer.

% Polymer	AUC (°/s)			
	PAM-4Na	PAM-18Na	PAM-18	HPMC
0%	23.14	342.70	289.20	113.70
10%	19.18	293.90	499.10	184.30
20%	14.20	515.60	585.70	161.70
30%	11.61	750.80	780.60	190.90
40%	10.70	816.50	720.30	306.10

**Table 6:** Values of dissolution efficiency (*DE*) for ampicillin trihydrate tablets at different proportions of polymeric materials at 37 °C, using two media for physiological simulation.

Media	% Polymer	Dissolution Efficiency Percentage (%)			
		PAM-4Na	PAM-18Na	PAM-18	HPMC
Gastric	0	87.69	87.69	87.69	87.69
	10	99.78	98.93	61.4	68.36
	20	99.27	53.91	65.71	34.51
	30	95.44	18.35	67.58	24.98
	40	93.91	12.52	53.96	18.13
Duodenal	0	93.87	93.87	93.87	93.87
	10	98.64	82.73	48.84	28.6
	20	100	16.77	51.13	16.39
	30	88.76	7.02	66.02	16.05
	40 *	89.07	6.61	62.53	11.42

The maximum dissolution time assessed was 45 min. * *p* < 0.05.

**Table 7:** Parameters and coefficients of determination of several kinetic models of dissolution.

Polymer	Media	% Polymer	Order Zero		Order 1		Higuchi		Korsmeyer–Peppas		
			*k_0_*	*R*^2^	*k*_1_	*R*^2^	*k_H_*	*R*^2^	*n*	*k_r_*	*R*^2^
PAM-4Na	Gastric	0	4.388	0.760	0.115	0.037	21.873	0.927	-	-	-
		10	10.662	0.917	0.461	0.976	34.196	0.997	0.108	1.283	0.624
		20	10.059	0.896	0.461	0.970	32.539	0.991	0.126	1.338	0.733
		30	7.167	0.913	0.326	0.968	28.605	0.990	0.224	1.674	0.678
		40	0.419	0.963	0.005	0.974	3.097	0.984	0.244	1.752	0.706
	Duodenal	0	7.791	0.899	−0.305	0.003	28.341	0.985	-	-	-
		10	10.517	0.859	0.461	1.000	34.502	0.980	0.060	1.149	0.549
		20	10.753	0.931	0.461	0.967	34.251	0.999	0.131	1.351	0.637
		30	3.516	0.711	0.206	0.927	20.721	0.929	0.172	1.486	0.768
		40	1.728	0.516	0.124	0.475	14.580	0.971	0.148	1.408	0.783
PAM-18Na	Gastric	0	4.388	0.760	0.115	0.037	21.873	0.926	-	-	-
		10	7.495	0.880	0.350	0.873	30.155	0.970	-	-	-
		20	2.419	0.996	0.095	0.827	16.885	0.909	1.280	1.194	0.988
		30	0.712	0.988	0.009	0.966	5.075	0.939	1.269	0.870	0.975
		40	0.419	0.963	0.005	0.964	3.097	0.983	1.256	0.754	0.974
	Duodenal	0	7.791	0.899	0.305	0.003	28.431	0.985	-	-	-
		10	4.121	0.890	0.211	0.937	22.167	0.962	-	-	-
		20	0.533	0.932	0.006	0.953	4.0163	0.993	1.353	0.823	0.963
		30	0.267	0.948	0.003	0.947	1.9177	0.916	0.964	0.656	0.972
		40	0.232	0.930	0.003	0.931	1.6897	0.926	1.006	0.618	0.972
PAM-18	Gastric	0	4.388	0.760	0.115	0.037	21.873	0.926	-	-	-
		10	0.779	0.821	0.021	0.892	7.528	0.916	0.279	1.900	0.958
		20	0.400	0.675	0.012	0.744	3.966	0.792	0.136	1.368	0.877
		30	0.204	0.739	0.012	0.785	1.978	0.834	0.062	1.154	0.905
		40	1.292	0.894	0.030	0.962	12.262	0.963	0.566	3.682	0.965
	Duodenal	0	7.791	0.899	0.305	0.003	28.431	0.985	-	-	-
		10	0.524	0.609	0.010	0.649	5.285	0.739	0.272	1.869	0.824
		20	0.361	0.640	0.007	0.683	3.602	0.763	0.163	1.457	0.849
		30	0.376	0.724	0.012	0.789	3.693	0.836	0.123	1.328	0.913
		40	0.786	0.867	0.023	0.943	7.512	0.947	0.267	1.848	0.975
HPMC	Gastric	0	4.388	0.760	0.115	0.037	21.873	0.926	-	-	-
		10	2.343	0.960	0.651	0.627	21.616	0.977	0.756	5.699	0.982
		20	1.172	0.998	0.020	0.989	10.602	0.976	0.663	4.600	0.980
		30	0.626	0.990	0.009	0.997	5.738	0.996	0.593	3.113	0.994
		40	0.525	0.988	0.006	0.992	4.803	0.988	0.577	3.779	0.998
	Duodenal	0	7.791	0.899	0.305	0.003	28.431	0.985	-	-	-
		10	0.695	0.986	0.010	0.979	6.209	0.941	0.418	2.620	0.898
		20	0.392	0.974	0.005	0.977	3.573	0.967	0.464	2.913	0.965
		30	0.467	0.948	0.006	0.950	4.179	0.909	0.516	3.284	0.902
		40	0.209	0.775	0.002	0.767	1.868	0.740	0.310	2.044	0.764

-, model not evaluated because of insufficient data.
